# Observation of elevated fasting blood glucose and functional outcome after ischemic stroke in patients with and without diabetes

**DOI:** 10.18632/oncotarget.19074

**Published:** 2017-07-07

**Authors:** Wen-Yu Xue, Yan-Cheng Xu, Yu-Wen Wu, Miao Yang

**Affiliations:** ^1^ Department of Endocrinology, Zhongnan Hospital of Wuhan University, Wuhan, China

**Keywords:** fasting blood glucose, ischemic stroke, functional outcome, stroke severity, Chinese

## Abstract

During May 2015 to October 2016, this prospective study enrolled a total of 438 patients with acute ischemic stroke(AIS), meanwhile, records regarding the severity of initial stroke and neurological outcomes at three months, as well as other examination were completed in patients on admission, as well as the measurement and evaluation of fasting blood glucose(FBG) levels. At admission, the median FBG levels in patients with a minor stroke (n=124), [P<0.001]) was significantly lower than that observed in patients with other degrees of stroke. The poor functional outcome distribution across the FBG quartiles ranged from 13.8 % (first quartile) to 59.6% (fourth quartile), with P <0.001. Compared with the reference category (first quartile), patients in the highest quartile had a relative risk of 3.12 (95% confidence interval [CI], 1.88-6.15; P<0.001) while those in the second and third quartiles had relative risks of 1.76 (95% CI, 1.21-3.03; P=0.035) and 2.23 (95% CI, 1.50-3.69; P=0.010), respectively. Furthermore, in the patients without diabetes, FBG level was observed to be increased and indicated an increased risk of disability (odds ratio [OR]: 1.30 (95%CI 1.13-1.61), P=0.002), however, similar result was not detected in patients with prior diabetes (P=0.089). In conclusion, elevated FBG levels after stroke may suggest poor functional outcome at 3-month in patients without a previous history of diabetes.

## INTRODUCTION

Diabetes is a metabolic disease characterized by hyperglycemia, which poses a great deal of harm to human health. By estimation, this disease confers about a two-fold excess risk for a wide range of cardiovascular disease (CVD), which is found to be independent from other conventional risk factors [1]. In a previous study of the same field, adults without known CVD or diabetes, an additional measurement of HbA1c values combined with conventional CVD risk assessment was suggested to be associated with none apparent effect on predicting the risk of CVD [2]. Furthermore, glycemia levels have been proved to be positively associated with the development progression of CVD [3], the involvement of information on glycemia measures might contribute to the improvements in CVD prediction capability [4–5]. In adults with hypertension of our country, patients with a fasting blood glucose (FBG) concentration ≥7.0mmol/l or diabetes always accompanied with higher risk of first stroke, which can be reduced by 34% with the application of folic acid treatment [6].

Hyperglycaemia is found to be prevalent in acute ischemic stroke (AIS) patients, accounting for 60% of patients at admission, both common in diabetes and non-diabetics populations [7]. Hyperglycemia following stroke may have adverse effect on the clinical outcome, which has been proved by increasing evidence. For example, previous studies had confirmed that final infarct volume [8], poor clinical outcome and mortality [9–10] are usually accompanied with hyperglycemia in stroke patients on admission. Meanwhile, a previous prospective cohort study also suggests the role of tighter glucose control in preventing the occurrence of primary stroke [11].

However the benefits of glucose monitoring and the value of FBG measurement for the prediction of functional outcomes in stroke patients with or without diabetes it still being debated. For instance, a study designed as a meta-analysis, on the basis of the extraction and analysis of past related studies, it was found that such investigation did not show any benefit from intensive control of blood sugar levels in patients after stroke [12]. With respect to the above, it was hypothesized in the present study that serum FBG levels could be associated with functional outcome in stroke patients with or without diabetes. Therefore, the study was carried on to determine the clinical significance of adding information on FBG to conventional cardiovascular risk factors assessment in predicting functional outcome in stroke patients with or without diabetes.

## RESULTS

From all the 602 patients, 461 patients were diagnosed with AIS (44 with onset of symptoms > 24hours, 23 with malignant tumor, 26 without informed consent, 15 with renal insufficiency, 12 with infection, 10 with history of brain trauma in past 3 months and 11 with blood samples lost were not analyzed) and 438 patients completed follow-up (15 lost to follow-up and 8 withdraw the experiment). However, baseline characteristics of all the 438 patients as well as the overall cohort were comparable [age (P=0.79), gender (P=0.52), BMI (P=0.61) and NIHSS score (P=0.39)]. FBG levels were obtained in those patients with a median value of 5.60mmol/l (IQR, 4.95–6.96mmol/l). Overall median age was 58 (IQR, 50-67) and 47.5% of them were female. Baseline characteristics of enrolled patients were described in Table 1. In addition, a sum up of 126 stroke patients were observed to combine with diabetes, among which 22.22% (28/126)patients received insulin treatment, and 59.5%(75/126) received hypoglycemic drugs.

**Table 1:** Baseline characteristics of patients with stroke ^ǂ^

	ALL	Poor outcomes	Good outcomes	Poor *vs*. Good outcomes	
N	438	150	288	OR(95%CI)	P ^ǂǂ^
Age, years medians (IQRs)	58(50-67)	64(57-73)	55(46-62)	1.55(1.20-1.77)	<0.001
Female, (%)	47.5	49.3	46.5	1.09(0.98-1.25)	0.075
Prior vascular risk factors, (%)					
Hypertension	61.0	70.7	55.9	1.26(1.03-1.55)	0.012
Diabetes	28.8	36.0	25.0	1.40(1.05-1.83)	0.016
Hypercholesterolemia	29.7	28.7	30.2	0.94(0.84-1.03)	0.202
Smoking	27.6	26.0	28.5	0.90(0.77-1.10)	0.403
Atrial fibrillation	23.3	31.3	19.1	1.76(1.40-2.39)	<0.001
Previous TIA	21.5	24.5	19.8	1.52(1.13-2.17)	0.116
Pre-stroke treatment, (%)					
Anti-platelet agents	29.2	28.0	29.9	0.91(0.75-1.04)	0.415
Anticoagulants	16.2	18.7	14.9	1.25(0.99-1.85)	0.503
Acute treatment, (%)	23.7	15.3	28.1	0.72(0.63-0.82)	0.002
NIHSS at admission, medians (IQR)	8(4-12)	10(7-15)	6(5-11)	1.16(1.11-1.20)	<0.001
Lesion volumes, ml median(IQR) ^ǂ ǂ ǂ^	25(11-36)	36(18-47)	21(8-30)	1.78(1.37-2.35)	0.001
Biomarkers, median(IQR)					
FBG, mmol/l	5.60(4.95-6.96)	6.62(5.85-7.86)	5.14(4.45-6.24)	1.29(1.15-1.45)	<0.001
Hs-CRP, mg/dl	0.55(0.22-1.49)	0.76(0.40-1.86)	0.48(0.16-1.05)	1.13(1.02-1.24)	0.016
HCY, umol/l	15.9(12.3-20.1)	18.8(14.3-24.2)	14.6(11.0-17.6)	1.08(1.01-1.22)	0.026

^ǂ^ A good functional outcome was defined as a mRS score of 0 to 2 points, while poor outcome was defined as 3-6 points.

^ǂǂ^ *P* value was assessed using Mann-Whitney U test or Chi-Square test.

^ǂǂǂ^ MRI with diffusion-weighted imaging (DWI) was available in some patients, N=349.

IQR, interquartile range; NIHSS, National Institutes of Health Stroke Scale; TIA, transient ischemic attack; Hs-CRP, high-sensitivity-C-reactive protein; HCY, Homocystinuria; FBG, fasting blood glucose.

In the comparison of FBG levels, no obvious statistical difference was found in those screened patients with or without diabetes (P=0.32). Spearman correlation analysis suggested that there was positive correlation between serum Hs-CRP and FBG(r=0.202, P<0.001). Meanwhile, FBG was also correlated with the infarct volume (N=349; r=0.186; P=0.001). At admission, in patients with a minor stroke (NIHSS score <5; n=124), median FBG level was lower than that in patients with moderate-to-high stroke [5.05(IQR, 4.69–6.14)mmol/l *vs*. 5.92(IQR, 5.36–7.43)mmol/L;P<0.001). Positive correlation between FBG concentration and NIHSS score was also found by applying Nonparametric Spearman rank correlation, indicating statistical difference (*r*=0.233; *P*<0.001).

**Figure 1:**
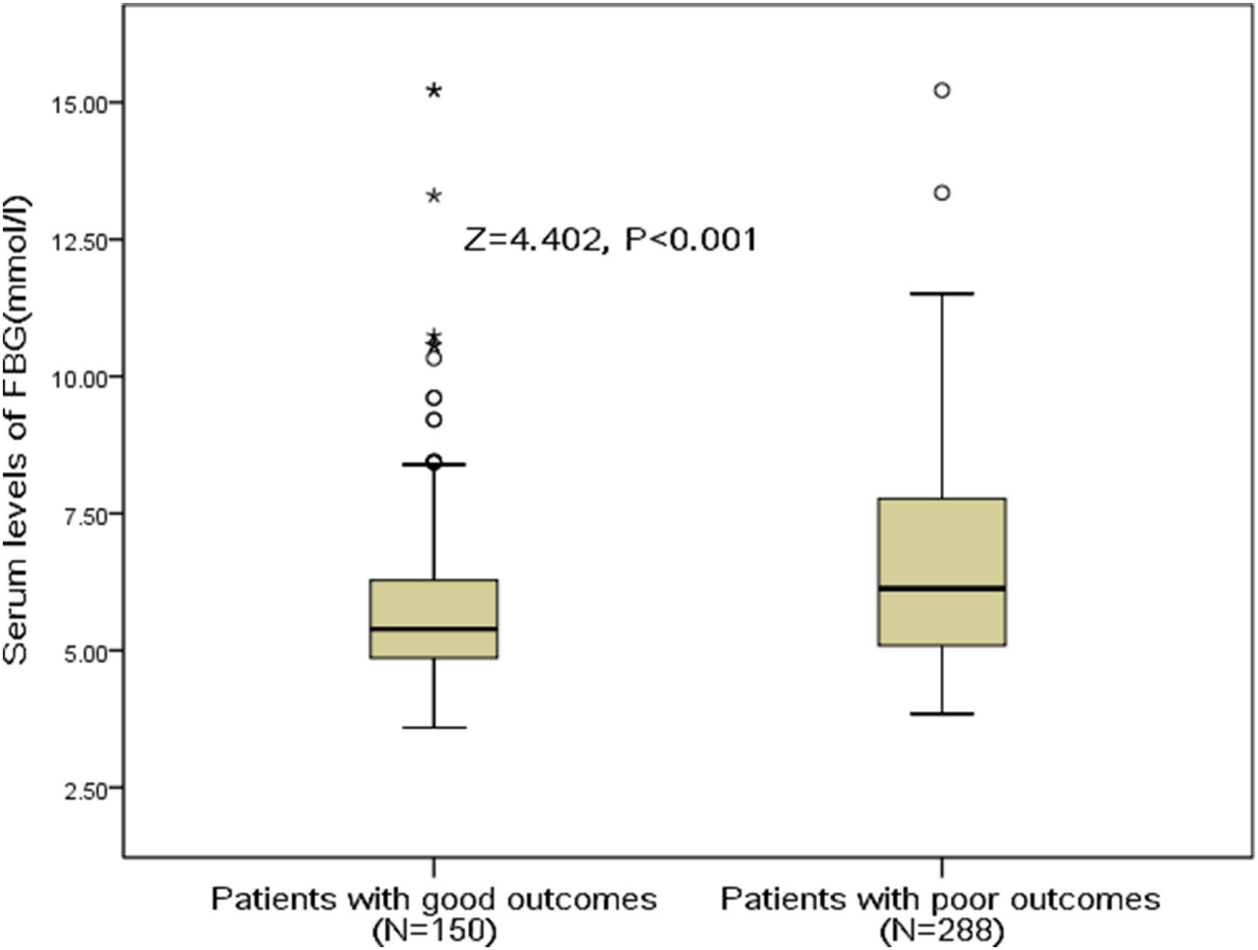
Serum FBG levels in stroke patients with good outcomes and poor outcomes A good functional outcome was defined as a mRS score of 0 to 2 points, while poor outcome was defined as 3-6 points. FBG, fasting blood glucose.

**Figure 2:**
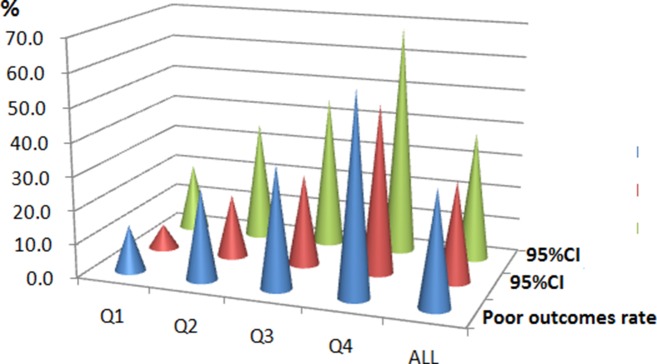
The prevalence of poor outcomes at 3 months after stroke onset according to the baseline FBG quartiles FBG levels in Quartile 1 (<4.95 mmol/l), Quartile 2 (4.95-5.60mmol/l), Quartile 3 (5.61–6.96mmol/l), and Quartile 4 (>6.96 mmol/l). The data present as the prevalence and 95%CI. A poor functional outcome was defined as a mRS score of 3 to 6 points. FBG, fasting blood glucose; CI, confidence interval.

In the follow-up period of 3 months, a poor functional outcome was found in 150 patients (34.2%; 95%CI: 29.8%-38.7%) with a median mRS score of 4 (IQR, 3–6). Furthermore, the mortality rate was 12.8% with 56 patients died in the experimental process (95%CI: 9.7%-12.9%). By comparison, serum FBG levels were detected to be much higher in patients with poor functional outcomes than those with good outcomes (6.13 [IQR, 5.09–7.78] vs. 5.39 [IQR, 4.86–6.29]mmol/l; Z=4.402; P<0.001; Figure 1.). The poor functional outcome distribution across the FBG quartiles ranged between 13.8 % (first quartile) to 59.6% (fourth quartile) (P<0.001; shown in Figure 2).

Univariate logistic analysis results were presented in Table 1. It was discovered that in the whole study populations, serum FBG levels was measured to be increased and was negatively associated with poor clinical outcome at 3 months (P<0.001). Other predictors of functional outcomes status consisted of age (P<0.001), NIHSS score (p<0.001), presence of hypertension (P=0.012), diabetes (P=0.016) and atrial fibrillation (P<0.001), acute treatment (P=0.002), lesion volumes (P=0.001), and serum levels of HS-CRP (P=0.016) and HCY (P=0.026), all of which showed statistical differences. For further determination of the connection between serum FBG levels and functional outcomes of patients, and following the adjustment of age, sex, NIHSS score, time from onset to blood collection, stroke syndrome, stroke etiology, pre-stroke treatment, acute treatment, vascular risk factors and serum levels of Hs-CRP and HCY, multivariate logistic regression was carried on afterwards in the whole subjects, which was shown in Table 2. Poor functional outcome was revealed to be correlated with elevated FBG levels in the serum (P=0.006), older age (P=0.008), higher NIHSS score (P<0.001), not received acute treatment (P=0.003), higher levers of HS-CRP and HCY. Furthermore, the risk of poor outcomes was significantly increased for patients in each of the 3 highest quartiles of the distribution of FBG. Compared with the preset reference category (first quartile), patients in the highest quartile had a relative risk of 3.12 (95% CI, 1.88-6.15; P<0.001), while those in the second and third quartiles had relative risks of 1.76 (95% CI, 1.21-3.03; P=0.035) and 2.23 (95% CI, 1.50-3.69; P=0.010), respectively. In the subgroup of patients (n=349) in whom MRI evaluations were performed, FBG was an independent poor outcome predictor with an OR of 1.28 (95% CI, 1.05 – 1.46; *P<*0.001) after adjustment for both lesion size [OR=1.43; 95%CI: 1.25-1.66; P<0.001] and the NIHSS score [1.15(1.04-1.29); P<0.001].

**Table 2:** Multivariate analysis of predictors of poor functional outcomes in stroke patients ^ǂ^

Predictors	OR	95% CI	P
Age (per unit increase)	1.50	1.21-1.76	0.008
NIHSS (per unit increase)	1.13	1.08-1.22	<0.001
Hypertension (yes vs. no)	1.18	1.02-1.45	0.069
Diabetes (yes vs. no)	1.29	0.94-2.03	0.208
Atrial fibrillation (yes vs. no)	1.50	0.84-3.12	0.627
Acute treatment (yes vs. no)	0.82	0.73-0.89	0.003
Stroke etiology (Vessel occlusive vs. other)	2.15	1.44-3.32	0.006
FBG (per unit increase)	1.21	1.07-1.38	0.006
HS-CRP (per unit increase)	1.10	1.02-1.19	0.011
HCY (per unit increase)	1.06	1.01-1.20	0.032

^ǂ^ Multivariable model included all of the following variables: age, sex, NIHSS score, time from onset to blood collection, stroke syndrome, stroke etiology, pre-stroke treatment, acute treatment, vascular risk factors and serum levels of Hs-CRP, HCY and FBG.

OR, odds ratio; CI, confidence interval; NIHSS, National Institutes of Health Stroke Scale; Hs-CRP, high-sensitivity-C-reactive protein; HCY, Homocystinuria; FBG, fasting blood glucose.

In stroke patients with diabetes, 54 patients (42.9%; 95%CI: 34.2%-51.5%) had poor outcomes; additionally, 96 out the 312 patients with the absence of diabetes (30.8%; 25.6%-35.9%) had poor outcomes (P=0.016). As illustrated in Tables 3 and 4, multivariate stratified logistic regression analyses demonstrated that in patients without diabetes, FBG levels was discovered to be increased and associated with an increased disability risk (OR1.30 (95%CI 1.13-1.61), P=0.002), however, similar results were not detected in patients with prior diabetes (P=0.089). Interestingly, functional outcome was strongly associated with age, NIHSS score, acute treatment, serum levers of HS-CRP and HCY in both subgroups (both P<0.05). In stroke patients with diabetes, patients in the highest quartile had a relative risk of 3.76 (95% CI, 2.33-7.15; P<0.001) compared with the reference category (first quartile), while those in the second and third quartiles had relative risks of 2.05 (95% CI, 1.36-3.19; P=0.032) and 2.55 (95% CI, 1.76-3.92; P=0.006), respectively. In the subgroup of patients (n=83) in whom MRI evaluations were performed, FBG was an independent poor outcome predictor with an OR of 1.49 (95% CI, 1.18 – 1.85; *P<*0.001) after adjustment for both lesion size [OR=1.53; 95%CI: 1.32-1.69; P<0.001] and the NIHSS score [1.18(1.07-1.35); P<0.001].

**Table 3:** Multivariate analysis of predictors of poor functional outcomes in stroke patients with diagnosis of diabetes

Predictors	OR	95% CI	P
Age (per unit increase)	1.42	1.18-1.61	0.011
NIHSS (per unit increase)	1.12	1.08-1.20	<0.001
Hypertension (yes vs. no)	1.17	1.02-1.40	0.062
Atrial fibrillation (yes vs. no)	1.46	0.87-3.00	0.605
Acute treatment (yes vs. no)	0.85	0.75-0.92	0.003
Stroke etiology (Vessel occlusive vs. other)	2.19	1.48-3.30	0.007
FBG (per unit increase)	1.05	0.97-1.58	0.089
HS-CRP (per unit increase)	1.13	1.06-1.24	0.010
HCY (per unit increase)	1.08	1.02-1.21	0.028

^ǂ^ Multivariable model included all of the following variables: age, sex, NIHSS score, time from onset to blood collection, stroke syndrome, stroke etiology, pre-stroke treatment, acute treatment, vascular risk factors and serum levels of Hs-CRP, HCY and FBG.

OR, odds ratio; CI, confidence interval; NIHSS, National Institutes of Health Stroke Scale; Hs-CRP, high-sensitivity-C-reactive protein; HCY, Homocystinuria; FBG, fasting blood glucose.

**Table 4:** Multivariate analysis of predictors of poor functional outcomes in stroke patients without diagnosis of diabetes ^ǂ^

Predictors	OR	95% CI	P
Age (per unit increase)	1.63	1.31-1.95	0.005
NIHSS (per unit increase)	1.16	1.10-1.24	<0.001
Hypertension (yes vs. no)	1.18	1.03-1.48	0.075
Atrial fibrillation (yes vs. no)	1.53	0.89-3.18	0.655
Acute treatment (yes vs. no)	0.80	0.71-0.85	0.002
Stroke etiology (Vessel occlusive vs. other)	2.12	1.40-3.25	0.005
FBG (per unit increase)	1.30	1.13-1.61	0.002
HS-CRP (per unit increase)	1.09	1.02-1.21	0.013
HCY (per unit increase)	1.05	1.01-1.22	0.036

^ǂ^ Multivariable model included all of the following variables: age, sex, NIHSS score, time from onset to blood collection, stroke syndrome, stroke etiology, pre-stroke treatment, acute treatment, vascular risk factors and serum levels of Hs-CRP, HCY and FBG.

OR, odds ratio; CI, confidence interval; NIHSS, National Institutes of Health Stroke Scale; Hs-CRP, high-sensitivity-C-reactive protein; HCY, Homocystinuria; FBG, fasting blood glucose.

## DISCUSSION

Low fasting glucose levels were negatively associated with increased mortality in the nondiabetic population, which has been proved in several studies [13]. Fasting glucose play an important predictive role of poor neurological outcomes in patients with AIS, which is suggested to be superior to random glucose and HbA1c in patients on admission [14]. However, this study was designed as a retrospective study, included patients were all diagnosed with mild to moderate initial stroke [NIHSS score: 4(IQR, 2-8)] [18]. Significantly different from the above study as a prospective cohort study, our study proved that elevated FBG level predicted poor outcome in patients after the onset of stroke, which is consistent with predecessors’ study, suggesting that hyperglycemia might be a critical or increased disability risks in AIS patients [9, 15–16]. More importantly, it was found in the current study that FBG correlated with functional outcome was found to be obvious in those patients without previous diagnostic history of diabetes, rather than in those diabetics’ populations.

Like the previous study, a meta-analysis supported the idea that stress hyperglycemia, which was measured to be frequent in critically ill patients, might forecast increased mortality risk and poor outcome in nondiabetic patients after stroke, but not in patients who were diagnosed with diabetes in clinical [17]. Another study also proved such relationship of blood glucose and mortality in nondiabetic populations [15]. Consistent with above data, Yao et al. [7] also confirmed such relationship in nondiabetics. However, some confounding factors were not included in the analysis process of multivariable logistical regression, such as syndrome and etiology of stroke, pre-stroke treatment and acute treatment, which should be taken into serious consideration [7]. In fact, those factors might have effect on the correlation between FBG levels and functional outcomes.

From the above findings, it was proposed in the present study that in AIS, hyperglycemia is having a detrimental effect on patients who were confirmed without diabetes; however, such relationship was not found inpatients. Similar to our results, Stead et al. [18] reported that a particularly poor prognostic outcome was found in hyperglycemia patients who had none prior history of diabetes, worse than that in patients with known diabetes and hyperglycemia. However, there were also different opinions, for example, no significant difference was found by the research carried out by Sun et al. [14], it was discovered in their study that the predictive power of glycemic measures for poor neurological outcomes showed none obvious difference in patients with or without diabetes. Possible reason for such difference among study might be that there were remarkable differences in the inclusion of experimental patients, definition of hyperglycemia, and outcome measures among multiples studies, even though the role of hyperglycemia have been reported for the estimation of poor neurological outcomes in AIS patients.

Of course, to be cautious, this observational study cannot make absolution results regarding the causal relationship between FBG and functional outcomes. Meanwhile, the role of FBG treatments in influencing the stroke outcomes cannot be determined at the same time. It is suggested the performance of an ongoing prospective randomized clinical trial with a large sample-size of AIS patients might be significantly valuable to provide optimal management approaches for hyperglycemia [19]. In addition, poor glycemic control (baseline HbA1c) prior to AIS might exert a predictive role in the increased stroke severity and unfavorable long-term functional outcome [20]. In this study, we found that FBG was associated with stroke severity (NIHSS score).

The mechanism of increased serum concentrations of FBG with poor outcome following an acute stroke is not yet established. One previous research documented that the association between hyperglycemia and poor functional outcome might reflect stress response rather than a deleterious effect of glucose [20], which is supported by our findings. Stress response may have an obvious role in induce the occurrence of hyperglycemia, especially at the acute phase of the disease [14]. Furthermore, stress was revealed to have close relationship with cytotoxic neurotransmitters, inflammation, and insulin resistance, all of those might result in higher risk of poor neurological outcomes in stroke and hyperglycemia patients, as well as rise in mortality [21–22]. A previous study has demonstrated the detrimental effect of hyperglycemia on the brain in an animal model [23]. Such deleterious effect of glucose on the damage to the brain was suggested to be even more serious after ischemia when compared to that in chronic hyperglycemia [23–24]. In addition, possible cause might also related to the following speculation, the acute hyperglycemia may stimulation glycation, which is a essential regulatory protein participated in fibrinolysis process, and then promote an abnormal thrombophilic state, indicating adverse effect of those patients in the late stage [25–26]. Lastly, by affecting mitochondrial function in the ischemic penumbra, hyperglycemia may directly result in cortical acidosis and cell death [27]. Subsequently, it may impair cerebrovascular reactivity condition in the microvasculature, disturbing reperfusion after recanalization [27]. Meanwhile, blood-barrier permeability may be altered by hyperglycemia; blood-barrier may thus be disrupted, which eventually aggravate brain edema formation, resulting in hemorrhagic transformation [28].

Strengths of our study include the fact that we collected data on a wide range of potentially confounding risk factors, such as age, sex, NIHSS score, time from onset to blood collection, stroke syndrome, stroke etiology, pre-stroke treatment, acute treatment, vascular risk factors and serum levels of Hs-CRP and HCY), which allowed us to estimate the independent effect of FBG. Second, different strategies were chosen using the FBG quartiles, and multivariate logistic regression analyses were further performed in accordance with the situation of previous history of diabetes. Lastly, this is a prospective cohort study, which was significantly different from that of previous retrospective study.

Several limitations of this study should be considered. Firstly, the value of glycemia measurement to screen for diabetes was not addressed to reduce diabetes-specific microvascular complications, nor relevant etiologic and therapeutic questions. Second, sample size of the study was relatively small, which may limit general applicability of this study. Before broad implementation, additional studies (Multi-center, large sample) are needed for external validation. Third, without serial measurement of circulating FBG, this study yielded no data regarding when and how long biomarkers were elevated in these patients. In addition, levels of HbA1c in the blood were not tested in our study, the association of HbA1c with FBG and stroke outcomes can thus not be determined in the present study. However, a previous study had suggested that HbA1c levels measured in type 2 diabetic patients with AIS on admission are highly correlated to functional outcomes of stroke patients [20]. Fourth, this study measured FBG in serum, not in cerebral spinal fluid (CSF). It is still uncertain whether peripheral FBG levels reflect similar changes in the central nervous system (CNS). Further study is needed to confirm the correlation between serum levels and CSF levels of FBG. Lastly, information on medication use was incomplete during the period of follow-up, which may have an influence in the estimation of the effect of individual risk factors, or risk models, on outcomes. In addition, causal relationship between FBG and stroke outcomes cannot be determined in this observational study. Meanwhile, it is difficult to know that treatment strategies were uniform for AIS patients with similar NIHSS scores and whether a variance may have affected outcomes.

To summarize, an elevated level of FBG after stroke might suggest poor functional outcome during the follow-up period within3 months, especially in patients without diabetes history. Whether FBG elevated should be eliminated in stroke patients to maintain their FBG concentrations optimal to prevent poor outcomes require to be verified by further long-term controlled clinical trials.

## MATERIALS AND METHODS

A prospective cohort study was performed at the emergency department of our hospital. From May 2015 to October 2016, consecutive first-ever AIS patients in China were identified and incorporated in the study. All patients were admitted in the hospital within 24 hours after the attack of a stroke. The definition of ischemic stroke was made according to World Health Organization recommendations, which was defined as a rapid occurrence of neurological deficit (both focal and global) of cerebrovascular cause, which persists beyond 24 hours or is interrupted by death within 24 hours [29]. The clinical diagnoses were validated on the basis of computed tomography (CT) and/or magnetic resonance imaging (MRI; blindly assessed by the XU YC). The diagnosis of type 2 diabetes was achieved by using WHO diagnostic criteria (glycated hemoglobin (HbA1c) level ≥ 6.4% or a fasting blood sugar (FBS) ≥ 6.11mmol/l) [30].

Exclusion criteria: (1) Patients with malignant tumor, (2) patients with renal insufficiency (creatinine>1.5 mg/dl), (3) patients with previous history of seizure disorder, brain trauma, disturbance of consciousness,(4) patients with acute and chronic infection that were confirmed by medical recording history; (5) patients who refused to included and lost blood samples. The present study has been approved by the ethics committee of the Zhongnan Hospital of Wuhan University. Study protocols were informed to all the included participants or their relatives, written informed consents were obtained prior to the performance of the research.

Clinical information of included patients was collected and recorded carefully. Firstly, general information including demographic data (age and sex), and history of risk factors (hypertension, diabetes mellitus, atrial fibrillation, hyperlipidemia and smoking habit) were obtained in those patients at admission. Other information such as pre-stroke therapy (anti-coagulants and anti-platelet agents) and acute treatment (IV thrombolysis and/or mechanical thrombectomy) was recorded. The severity of stroke was assessed by using the National Institutes of Health Stroke Scale (NIHSS) in those patients on admission. Strokes were classified based on Trial of Org 10172 in Acute Stroke Treatment (TOAST) classification criteria, and clinical stroke syndrome was determined applying the criteria of Oxfordshire Community Stroke Project (OCSP). MRI with diffusion-weighted imaging (DWI) was available in some patients enrolled the study. The following formula was used for the calculation of infarct volume, and the volume= 0.5×*a*×*b*×*c* (*a*: maximal longitudinal diameter, *b*: the maximal transverse diameter perpendicular to *a*; *c*: the number of 10-mm slices containing infarct) [31].

Functional clinical outcomes were obtained on a time range of 3 months after the onset of stroke onset according to the modified Rankin Scale (mRS) [32], these results was blinded to blood biomarkers levels. Via a follow-up telephone interview, the evaluation of functional outcomes was performed by one trained medical staff with the patient or with the relative. The primary end point of this study was good functional outcome of stroke patient, with the mRS score ranging from 0 to 2 points. Secondary end point was predefined as death induced by any cause within a 3 months follow-up period. Under fasting state in the early morning, involved patients received blood samples collection on the first day of admission. Serum levels of FBG, high-sensitivity-C-reactive protein (Hs-CRP) and Homocystinuria (HCY) were measured using routine laboratory methods. In this study, the intra-assay coefficient of variation [CV] and inter-assay CV of FBG were 1.5-2.4% and 1.8 %-2.9%, respectively.

Data collected from the study was expressed as percentages for categorical variables and as medians (interquartile ranges, IQRs) for continuous variables. Comparison between groups was using Mann-Whitney U test and chi-square test. Bivariate correlation was analyzed with Spearman's Rank correlation. Furthermore, a binary logistic regression model was involved to analyze the influence of FBG levels in functional outcome of stroke patients after admission, allowing adjustment for confounding factors (age, sex, NIHSS score, time from the onset of stroke to blood collection, syndrome and etiology of stroke, pre-stroke treatment, acute treatment, vascular risk factors and serum levels of Hs-CRP and HCY). Following statistical analysis, all results were expressed as adjusted odds ratios (OR) with the corresponding 95% Confidence interval (CI). For a more detailed exploration of potential association between FBG levels and functional outcome of patients, multivariate analysis models were used to estimate adjusted OR and 95% CIs of functional outcomes for FBG quartiles (with first FBG quartile as reference). Furthermore, a multivariate logistic regression analysis was performed on the basis of patients with and without prior history of diabetes. All statistical data were analyzed using the SPSS for Windows, version 20.0 (SPSS Inc., Chicago, IL, USA). Statistical significance was defined as p < 0.05.

## Abbreviations

CVD: cardiovascular disease
FBG: fasting blood glucose
AIS: acute ischemic stroke
CSF: cerebral spinal fluid
CNS: central nervous system
CT: computed tomography
MRI: magnetic resonance imaging
HbA1c: Glycated hemoglobin
FBS: fasting blood sugar
NIHSS: National Institutes of Health Stroke Scale
TOAST: Trial of Org 10172 in Acute Stroke Treatment
OCSP: Oxfordshire Community Stroke Project
DWI: diffusion-weighted imaging
mRS: modified Rankin Scale
Hs-CRP: high-sensitivity-C-reactive protein
HCY: Homocystinuria
IQR: interquartile ranges
OR: odds ratios
CI: confidence interval
